# The effectiveness of community-based interprofessional education for undergraduate medical and health promotion students

**DOI:** 10.1186/s12909-024-05066-1

**Published:** 2024-01-26

**Authors:** Chawin Suwanchatchai, Kitsarawut Khuancharee, Suthee Rattanamongkolgul, Kittipong Kongsomboon, Manasvin Onwan, Anantapat Seeherunwong, Pacharapa Chewparnich, Piyanuch Yoadsomsuay, Pattakorn Buppan, Ormjai Taejarernwiriyakul, Sirikul Thummajitsakul, Pimonporn Chaovipoch, Sunisa Krainara, Pariyakorn Sanguankittiphan, Rattiporn Kosuwin, Pakarang Srimee, Yuparat Odglun, Supim Wongtongtair

**Affiliations:** 1 Department of Preventive and Social Medicine, Faculty of Medicine, Srinakharinwirot University, Nakhon Nayok, Thailand; 2 Department of Health Promotion, Faculty of Physical Therapy, Srinakharinwirot University, Nakhon Nayok, Thailand

**Keywords:** Community-based interprofessional education, Interprofessional education, Undergraduate students

## Abstract

**Background:**

Community-based interprofessional education (CBIPE) has been proven effective in enhancing the interprofessional competencies of medical and health professional students. However, there is a lack of evaluation on the impact of experiential CBIPE among undergraduate medical and health promotion students in Thailand. Therefore, the objective of this study is to assess the influence of CBIPE learning on the collaborative competencies of these students.

**Methods:**

A one-group pre-posttest design in 193 (152 medical students and 41 health promotion) students were involved in the CBIPE program, later divided into 12 groups. Data was collected by direct observations of mentors using the Interprofessional Collaborative Competencies Attainment Survey (ICCAS). The Wilcoxon matched-pairs signed-rank test was conducted to evaluate the effectiveness of the CBIPE program.

**Results:**

A total of 175 (90.67%) completed ICCAS and satisfaction questions before and after the CBIPE program. The mean age of respondents was 20.29 ± 1.63 years; 60.57% were women and 39.43% were men. The results showed a significant increase in collaborative competencies before and after the 2-week course. Gender-stratified analysis showed an improvement after CBIPE training for all subscales in women, while the communication, collaboration, conflict management, and functioning team skills segment score was significantly higher in the post-assessment among men.

**Conclusion:**

The implementation of CBIPE learning was successful in enhancing collaborative competencies among both medical and health promotion students. These findings will provide valuable insights for the design and improvement of CBIPE learning programs in other universities.

## Introduction

Community-based education (CBE) is one of the best approaches for facilitating collaborative skills worldwide in health professions students [1, 2]. CBE learning activities often utilize the community as a significant learning environment. In which not only students but also teachers, mentors, community people, and representatives of other government sectors are also engaged throughout the educational experience and fieldwork practical [3]. Community-based interprofessional education (CBIPE) is a collaborative learning approach that takes place in a community setting. It involves a group of students with diverse educational backgrounds coming together to learn and work on projects. This method encourages teamwork and provides opportunities to apply knowledge in real-life situations. The goal is to help learners gain practical experience and enhance their understanding of their respective fields within a community context [4, 5]. Therefore, the aspect of CBIPE learning is an interactive learning experience with direct professional cooperation. Previous studies have indicated that utilizing interactive interprofessional education (IPE) techniques, such as CBIPE learning, can effectively improve the cooperative abilities of health professional students [1, 5–11]. CBIPE's programs offer students a unique opportunity to delve into various concepts related to family medicine, primary care, social determinants of health, and cultural competence that are not typically covered in most health professional curricula [12, 13]. Moreover, the CBIPE program also helps motivate social accountability among health profession students [14]. The nature of CBIPE learning activities is mainly to provide healthcare services in rural and primary healthcare settings [5, 7, 15] and specific community contexts [11, 16, 17] were commonly used in Western countries [6, 8, 10, 18–20]. Although CBIPE learning programs have been implemented worldwide, there seem to be few reports on the implementation and results of the effectiveness of these programs in Southeast Asian contexts [9, 21]. Meanwhile, CBIPE learning in community contexts has no study and reports in Thailand, there seem to be few reports on the implementation and results of the effectiveness of IPE programs in healthcare settings [22–24]. The CBIPE learning program for medical and health promotion students has not yet been tested and published in Thailand. To ensure that medical and health promotion students in Thailand have a well-rounded set of Interprofessional Education (IPE) skills, including the ability to diagnose health issues within the community and develop and implement health-related innovations, a comprehensive CBIPE learning program is necessary. Therefore, we aimed to develop CBIPE practical learning and to evaluate the effect of CBIPE learning on collaborative competencies among undergraduate medical and health promotion students, at Srinakharinwirot University, Thailand.

## Methods

### Study design and population

**Fig. 1:**
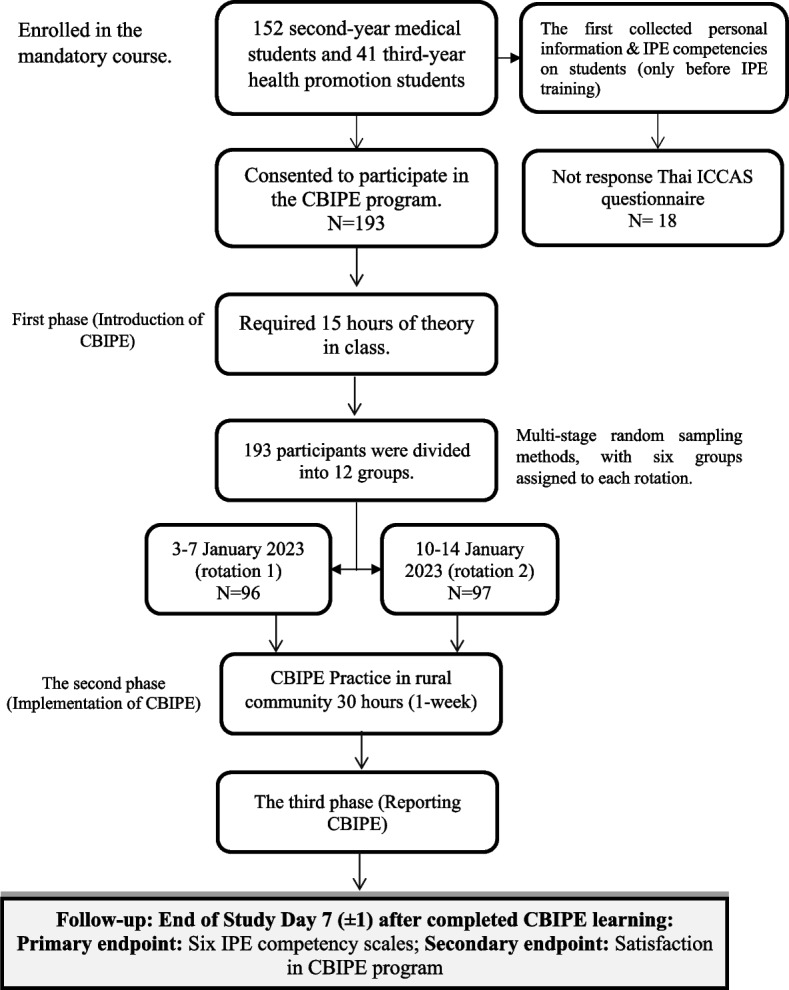
Study schema

**Fig. 2:**
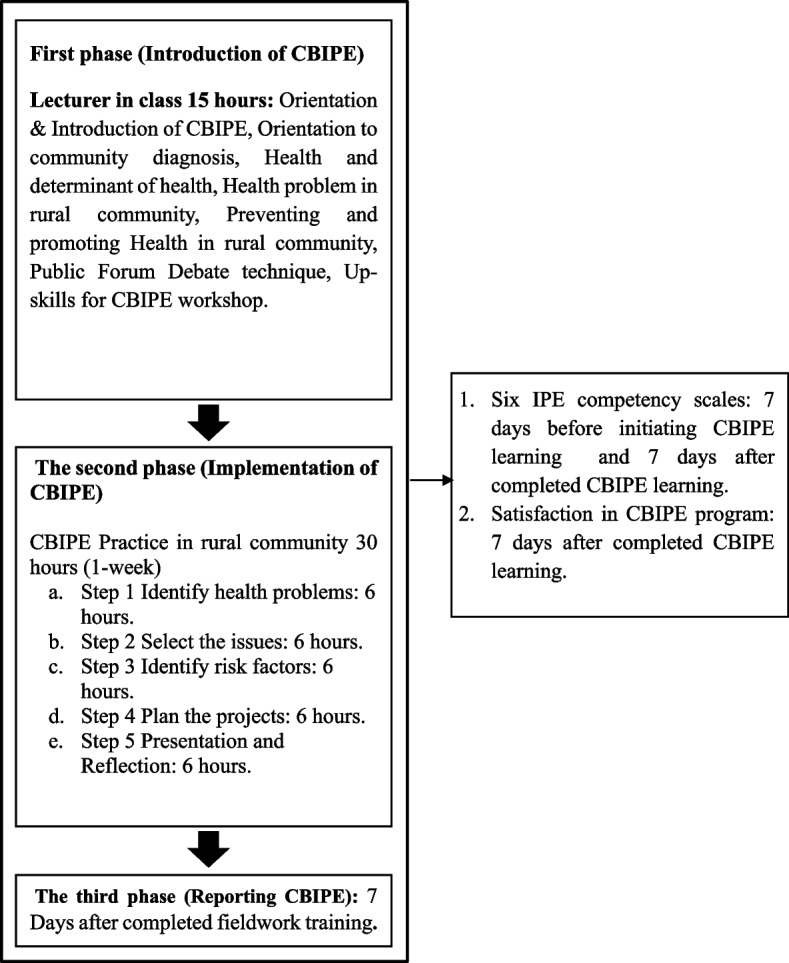
Conceptual framework and timeline for action on the CBIPE learning program

A single-group, non-randomization with a pre-post-posttest design was conducted to evaluate the development of collaborative skills (Fig. 1). The study involved a sample of 193 undergraduate students, comprising 152 s-year medical students and 41 third-year health promotion students. The target population enrolled in the mandatory course “Community Health Diagnosis through CBIPE learning program”, which required 15 h of theory in class and 30 h of fieldwork practice in the rural community of Ban Phrao subdistrict, Ban Na district, Nakhon Nayok province, Thailand over one week. The fieldwork took place during two separate rotations: from 3–7 January 2023 (rotation 1) and 10–14 January 2023 (rotation 2). The participants were divided into 12 groups using multi-stage random sampling methods, with six groups assigned to each rotation. Each group consisted of a minimum of 10 medical students and 3–4 health promotion students. All students were asked to complete the ICCAS questionnaire before and at the end of the course. The training program (First phase) started in December 2022 and data collection was completed in January 2023. IPE has been recently defined by the World Health Organization (WHO) as “occasions when two or more professionals learn with, from, and about each other to improve collaboration and the quality of care” [3]. We attempted to develop the CBIPE program based on the framework for action on interprofessional education and collaborative practice from the WHO and a thorough literature review. A conceptual framework and timeline for action on the CBIPE learning program are described in Fig. 2.

### CBIPE program description

#### First phase (Introduction of CBIPE)

During the initial week of the CBIPE program, participants were provided with comprehensive explanations regarding Interprofessional Education (IPE) and the program structure at Srinakharinwirot University. The curriculum includes lecture courses that incorporate small group discussions and simulations. The lectures will cover topics such as the context of community and cultural problems in rural communities, as well as the ethics of conducting surveys. Following the IPE skills training, students were divided into 12 groups and assigned to different villages within the rural community. Each group is supported by five associate teachers, consisting of one public health technical officer/nurse and four village health volunteers. The role of these associate teachers is multifaceted: (1) to contribute their professional expertise to enhance the quality of community fieldwork practice under the guidance of supervisors, (2) to assist students in comprehending and applying the knowledge acquired during community fieldwork practice, addressing any difficulties they may encounter, (3) to facilitate engaged learning by promptly responding to student inquiries, facilitating effective small group discussions, providing supplementary explanations or resources as needed, and introducing students to emerging issues in contemporary professional practice, and (4) to offer personalized academic guidance and support, including advice on collaborative skills and assessment. All supervisors underwent a three-day intensive training seminar for a small group, in-depth learning experience on how to promote engaged CBIPE before offering academic guidance, advising students on collaborative skills, and conducting assessments. The training aimed to provide them with the essential knowledge and skills to support students effectively in their CBIPE activities. The supervisor’s training program was conducted over three full days, on September 13th, 20th, and 27th, 2022.

#### The second phase (Implementation of CBIPE)

During the designated period, all students were allocated to a community for a duration of one week. This phase of their education is crucial as it enables students to develop an understanding of the distinct responsibilities and obligations associated with various professions. Additionally, it allows them to effectively assign tasks based on the specific roles and levels of authority within their respective fields. To actively engage with the local community, it is imperative for students to demonstrate respect for the cultural norms and values prevalent in the area. This approach facilitates effective communication and collaboration with community members, particularly in the context of healthcare initiatives. Under supervision, the students followed a structured five-step process to successfully implement the CBIPE Program, as outlined below:

- *Step 1 Identify health problems:* The initial phase involved the identification of individuals who hold prominent positions within the community, including the community leader, healthcare volunteer leader, and religious leader. Subsequently, the students conducted a survey to assess the health issues prevalent in the community, utilizing data from various sources such as local census records, historical records of morbidity and mortality, and the economic profile of the community. This data was then analyzed in order to diagnose the primary health problems affecting the community.
- *Step 2 Select the issues:* This step conducts a health-related needs assessment. The students would perform assessments and reports of the holistic aspects of the community health problems (community syndrome), which are biological, Social determinants, and community regulation. Then, prioritizing needs with community people. However, the identification of problems should be based on all perspectives and by both professions (medical and health promotion) and community people. In this step, the students would learn the community’s strengths, needs Informal and formal interviews, focus and concerns, ability to come together for change, and local leadership in the community.
- *Step 3 Identify risk factors:* The students conducted an analysis and presented findings on the biological and social factors that impact health. The students used the web of causation as a framework to explain the interrelationship between several disease-causing or risk factors that contribute to the cause of a specific medical condition in rural communities. This model helped them identify the risk factors that impact health in those communities.
- *Step 4 Plan the projects:* After conducting a thorough assessment of the community's needs, the students compiled a list of health problems that were identified as a result of the existing gap in health status. They then presented a comprehensive analysis of the web of causation in relation to these health problems. Subsequently, the students established goals, devised strategies, set objectives, and formulated project plans and health innovations in collaboration with the community members to address these problems. The proposed activities, content, and schedules of the projects and innovations were subject to prior discussion with the group's field supervisor and the healthcare professionals responsible for community healthcare services. Each group implemented their respective project or innovation, which spanned a duration of two weeks following the community diagnosis. Furthermore, during the community forum activities, each group evaluated and devised promotive and preventive initiatives targeting major non-communicable diseases such as diabetes mellitus and hypertension, with the aim of enhancing self-awareness regarding the prevention and management of complications associated with these diseases. These activities not only helped patients, but also served to educate the community on matters of health literacy.
- *Step 5 Presentation and Reflection:* In a formal setting, each group presents the outcomes of their community diagnosis and proposals for projects or innovations to a forum. This forum is attended by field supervisors, the head or staff from the local public health center, village health volunteers, and community leaders. The proposed projects or health innovations may take the form of initiatives aimed at improving health literacy within the community, collaborating with community members on disease prevention efforts, providing training for village health volunteers, or creating a supportive environment, among other possibilities. During this presentation phase, the groups not only discuss the findings of their community diagnosis and project proposals, but also engage in reflection on their interprofessional education (IPE) skills. For instance, students may share their personal experiences, discuss any unfinished plans, identify limitations encountered, and provide recommendations for future actions. These reflections take place within the interprofessional group, with guidance from the group's field supervisor.

#### The third phase (Reporting CBIPE)

By the end of the program, each group generated comprehensive reports through the documentation and reflection of their learning activities in the context of CBIPE.

### Data collection

Before implementing the CBIPE program, all participants were assessed for IPE competencies. At the end of the CBIPE program, all participants were given the post-test survey following the conclusion of the program. One supervisor was recruited and assessed the student’s IPE competencies by direct observation of student activities on day 7 (at baseline, before initiating CBIPE learning) and 7 days after completing CBIPE learning. To assess IPE competencies, we used the Interprofessional Collaborative Competencies Attainment Survey (ICCAS Thai version). The Thai version of the ICCAS was validated with an alpha Cronbach test score of 0.81 [22]. In this present study, the principal investigator trained the supervisor on how to observe and interpret each of the items of the Thai ICCAS in the first phase (Introduction of IPE). Moreover, we carried out a qualitative study performing a semi-structured questionnaire. A total of 12 semi-structured online questionnaires were conducted and asked to reflect on their enablers and barriers for CBIPE training. The questionnaire had an average duration of 30–45 min. All open-ended questions were gathered in January 2023. The qualitative semi-structured questionnaire's results, with 13 experts, were sent to all experts as a written summary. A written summary with the results was sent to all experts by e-mail. The results were used as a basis for the group discussion to clarify specific aspects of both semi-structured questionnaires.

### Data analysis

The data collected were analyzed using STATA version 14. Descriptive statistics were used for all variables. Six IPE competence scores were calculated and reported with their respective mean and standard deviation. The authors conducted the Wilcoxon matched-pairs signed-rank test to assess the efficacy of the CBIPE program. To assess the normality of each subscale, the Kolmogorov–Smirnov test was used. Analyses were subsequently stratified by gender. A statistical power analysis was conducted using Cohen's d effect size, with 0.2, 0.5, and 0.8 considered as small, medium, and large effect sizes respectively. An effect size greater than 1.00 indicates a statistically significant and strong intervention effect, while an effect size ranging from 0.51 to 1.00 suggests a statistically significant and moderate intervention effect. Statistically significant findings were determined by conducting two-sided analyses with a p-value below 5%. The qualitative data was analyzed using a conventional approach to content analysis [23]. The data was broken down into meaningful units and labeled appropriately. Based on their similarities and differences, the labeled units were grouped into subcategories and categories through comparative analysis. The categories and subcategories were examined and revised by the researcher, along with the extracted codes.

## Results

Out of the 193 students who participated in the CBIPE program, the majority were women at 60.57%, with an average age of 20.29 ± 1.63 years. 175 students, equivalent to 90.67%, provided responses to the ICCAS and satisfaction questions before and after completing the program. Unfortunately, 18 students were unable to complete the pre-posttest.

Table 1 shows the scores for the IPE competency scale for medical and health promotion students before and after following their participation in the CBIPE program. A Wilcoxon signed-rank test showed that there is a significant difference in the six domains of the IPE competence scale after the CBIPE program as compared to before. After undergoing CBIPE training, both men and women showed improvement in various skills related to communication, collaboration, conflict management, and functioning as a team. However, a gender-stratified analysis revealed that men had a significant improvement in these skills, while women showed improvement in all subscales. Among the subscales, only women showed improvement in subscales 3–4, which are "Roles and Responsibilities" and "Collaborative Family-Centered Approach" (Table 2). The satisfaction survey results revealed that most students were satisfied with the CBIPE program, with an average score of 4.39 ± 0.52 out of 5 points, as shown in Table 3.

**Table 1:** Mean scores of the subscales of the IPE assessment for medical and health promotion students before and after their training

Competency	Pre-test	Post-test	*P*-value	Cohen's d
1. Communication				
- Median(Range)	4.20(1–5)	4.60(3–5)	< 0.0001^*^	0.60
- Mean(SD)	4.15(0.72)	4.53(0.51)		
2. Collaboration				
- Median(Range)	4.33(1–5)	4.67(2–5)	< 0.0001^*^	0.44
- Mean(SD)	4.23(0.77)	4.54(0.57)		
3. Roles and Responsibilities				
- Median(Range)	4.50(1–5)	4.75(3–5)	< 0.0001^*^	0.47
- Mean(SD)	4.27(0.67)	4.55(0.51)		
4. Collaborative Family-Centered Approach				
- Median(Range)	4.33(1–5)	4.67(2–5)	0.0001^*^	0.33
- Mean(SD)	4.20(0.76)	4.43(0.60)		
5. Conflict Management/Resolution				
- Median(Range)	4.67(1–5)	5(3–5)	< 0.0001^*^	0.46
- Mean(SD)	4.47(0.65)	4.74(0.46)		
6. Team Functioning				
- Median(Range)	4.50(1–5)	5(2–5)	< 0.0001^*^	0.52
- Mean(SD)	4.23(0.67)	4.56(0.57)		

*IPE* Interprofessional education, *SD* Standard deviation

**Table 2:** Mean scores of the IPE subscales for medical and health promotion students before and after training, categorized by gender

Competency	Pre-test	Post-test	*P*-value	Cohen's d
**Males (*n* = 69)**				
1. Communication				
- Median(Range)	4.20(1–5)	4.60(3–5)	0.0005^*^	0.53
- Mean(SD)	4.10(0.82)	4.47(0.55)		
2. Collaboration				
- Median(Range)	4.33(1–5)	4.67(2–5)	0.0046^*^	0.23
- Mean(SD)	4.16(0.85)	4.46(0.67)		
3. Roles and Responsibilities				
- Median(Range)	4.25(1–5)	4.50(2–5)	0.1592	0.39
- Mean(SD)	4.22(0.77)	4.38(0.58)		
4. Collaborative Family-Centered Approach				
- Median(Range)	4.33(1–5)	4.67(2–5)	0.0533	0.26
- Mean(SD)	4.22(0.83)	4.41(0.65)		
5. Conflict Management/Resolution				
- Median(Range)	4.67(1–5)	5.00(3–5)	0.0027^*^	0.40
- Mean(SD)	4.40(0.75)	4.66(0.56)		
6. Team Functioning				
- Median(Range)	4.50(1–5)	5.00(2–5)	0.0006^*^	0.46
- Mean(SD)	4.20(0.80)	4.54(0.67)		
**Females (*n* = 106)**				
1. Communication				
- Median(Range)	4.20(2–5)	4.80(3–5)	< 0.0001^*^	0.67
- Mean(SD)	4.18(0.64)	4.56(0.48)		
2. Collaboration				
- Median(Range)	4.33(2–5)	4.67(3–5)	0.0001^*^	0.69
- Mean(SD)	4.28(0.71)	4.59(0.50)		
3. Roles and Responsibilities				
- Median(Range)	4.50(2–5)	4.75(3–5)	< 0.0001^*^	0.49
- Mean(SD)	4.30(0.61)	4.67(0.42)		
4. Collaborative Family-Centered Approach				
- Median(Range)	4.33(2–5)	4.67(2–5)	0.0005^*^	0.38
- Mean(SD)	4.19(0.72)	4.44(0.56)		
5. Conflict Management/Resolution				
- Median(Range)	4.67(2–5)	5.00(3–5)	< 0.0001^*^	0.53
- Mean(SD)	4.53(0.58)	4.79(0.38)		
6. Team Functioning				
- Median(Range)	4.50(3–5)	4.75(3–5)	< 0.0001^*^	0.58
- Mean(SD)	4.26(0.59)	4.58(0.49)		

*IPE* Interprofessional education, *SD* Standard deviation

**Table 3:** the levels of satisfaction observed among participants of the CBIPE program

Items	Mean ± SD	Satisfaction Levels
1. Understanding the value of IPE	4.43 ± 0.73	Satisfied
2. Understanding the roles and responsibilities of various health professionals is essential	4.47 ± 0.69	Satisfied
3. Understanding the competency required for collaboration and communication	4.44 ± 0.72	Satisfied
4. Understanding the importance of teamwork	4.31 ± 0.84	Satisfied
5. Implementing the community diagnosis program in real-life situations was deemed desirable	4.59 ± 0.62	Highly satisfied
6. The overall CBIPE program was well-designed	4.46 ± 0.68	Satisfied
7. The purpose of the program was clearly explained to the students	4.35 ± 0.82	Satisfied
8. The instructors played a role in promoting collaboration among students	4.59 ± 0.71	Highly satisfied
9. The number of instructors featured an adequate number of students	4.56 ± 0.68	Highly satisfied
10. The CBIPE program featured an adequate amount of running time	3.72 ± 1.14	Satisfied
11. Overall satisfied with the CBIPE program	4.39 ± 0.52	Satisfied

*CBIPE* Community-based interprofessional education, *IPE* Interprofessional education, *SD* Standard deviation

Most students openly reflected and explained that the CBIPE program helped them fill their knowledge gaps and also improved the IPE competency skills included improved competency awareness in collaborative leadership, communication skills, interprofessional conflict-solving skills, understanding the roles of other professionals, and understanding my roles within the collaborative practice. A participant from medical and health promotion students commented:

“I want to say that the CBIPE program helped me understand … and improved competency awareness in collaborative leadership, communication skills, interprofessional conflict-solving skills, understanding the roles of other professionals, and understanding my roles within the collaborative practice.” (Male medical student).

“Rural community is a good place to learn CBIPE skills; we learn how professionals communicate with patients, engage in discussions with students of other majors, and other professionals in a real situation.” (Female health promotion student).

“The impact of CBIPE program keeps us together to create health innovation sustainable for susceptibility population in rural community.” (Female medical student)

“These shared CBIPE fieldwork practices made me interested in my discipline and made me feel useful… I feel there was better communication between me and the medical students and I felt more comfortable with them” (Male Health Promotion student).

“IPE promotes skills and competences to work effectively in an interprofessional team. Improved communication enhances coordinated interprofessional collaborative practice…. improves the acceptance and thus the mutual understanding of both professions.” (Female medical student).

However, Students also reflected that limitations of the CBIPE program included insufficient running time and room for improvement when it came to preparing for public hearing forums.

“… It makes me so happy when I see my discipline’s effectiveness in a team. It’s too bad, there is not enough time for improvement when it comes to preparing for public hearing forums.” (Female health promotion student).

"Rural communities provide excellent opportunities to gain inter-professional skills in a real-world setting …. I felt that the program lacked sufficient running time and space for the preparation of public hearing forums." (Male medical student).

## Discussion

The present results have shown that the six domains of collaborative competence skills had a significant difference between the measurements before and after the CBIPE program. Gender-stratified analysis (Table 2), women showed improvement in all subscales, while men exhibited a higher score in the communication, collaboration, conflict management, and functioning team skills segment after the program. However, there was no significant difference in the collaborative approach centered on the patient and family and roles and responsibilities segment score. The students reflected that the CBIPE learning helped their competency in collaborative skills. This remarkable achievement was made possible through a cooperative strategy in CBIPE education. This strategy required regular and thorough interprofessional participation, gatherings, and conversations regarding creating health innovations. While the CBIPE learning endeavor, it became evident that the medical and health promotion students frequently convened to confer and work together on various health projects and novel initiatives. This concerted, cooperative undertaking proved to be both rigorous and fruitful. This present result supports the findings of other studies that reported improvements in collaborative skills through participation in CBIPE learning. The impact of CBIPE learning has shown that only the competence of the communication and functioning team had a moderate effect. Similar previous studies [5, 9, 24–27] indicate that heightened interprofessional discussion and communication within a designated course or learning activity can effectively increase awareness and facilitate a more cohesive teamwork environment in patient healthcare. Meanwhile, the four competencies, which include a collaborative approach focused on patients and their families, collaboration, clear roles and responsibilities, and conflict management, had a small effect. It is assumed that the program had only a small effect on enhancing competencies. Some studies similarly have pointed out that challenges arise in implementing IPE such as patient safety and a collaborative approach that focuses on the patient and their family [28]. The students in this study had limited exposure to collaborative work in health prevention and promotion, making it more difficult for them to appreciate the importance of patient/family-centered care. Their lack of real-world patient care experience may have contributed to this challenge in undergraduate students. However, This CBIPE program may be an effective approach to enhancing intrinsic motivation in health promotion and wellness in rural communities. The CBIPE program is designed to enhance the collaborative competencies of medical and health promotion students. It provides opportunities for active engagement of both students and community members in various learning activities throughout the educational experience [5–11]. The CBIPE program helps medical and health promotion students gain experience by working within the community and conducting community diagnosis. Community-based integrated care starts with holding community care meetings. During these meetings, the principles and goals of community-based integrated care are shared between different disciplines, and the roles of team members are clarified. In addition, individual care meetings are held where team members discuss the multidisciplinary team’s responses to a particular case. The team members try to reach a consensus on the problems patients are facing and possible solutions through polite discussions. Building good communication is essential to establish a base of consensus and shared principles. The promotion of interdisciplinary cooperation in community-based integrated care can be compared to the team-building process in Beckhard’s model [29]. Interprofessional teams work together to promote health and prevent illness in rural communities, even with limited timing and resources. However, improving skills such as active listening, conducting thorough interviews, making informed decisions, solving complex problems, and displaying effective leadership remains a challenge. Therefore, it is important to prioritize the enhancement of these skills for medical and health promotion students in the future. In our recommendations for future study, we suggest that the CBIPE curriculum should focus on promoting collaborative family-centered care for both undergraduate and postgraduate health professional education. The present study also revealed that most of the students were satisfied with the CBIPE program. Most students openly reflected that the CBIPE program's strengths were its ability to facilitate discussions with students from different majors, group mentors, and community members. They found it valuable to connect with others' perspectives and learn about the roles of others in a participation-centered program. However, most students reflected that the CBIPE program had limitations, particularly in terms of insufficient time for improvement when it came to preparing for public hearing forums.

Our present study had some limitations. First, the present study did not have a comparison group, which is an essential component of any study. To validate the assessment of the changes in collaborative skills, we suggest that future studies with longer durations should incorporate a comparison group. Further analysis of clinical trials may help identify the CBIPE program's effect on IPE competence. This would provide more robust evidence of the effectiveness of the CBIPE approach. Second, Collaborative skills were assessed before and after observation. Although our results are promising, the long-lasting effect of the CBIPE program was not measured. Therefore, the mean change in collaborative skills could be observed and assessed over a more extended period, such as follow-up assessments immediately, at 30 days, and 6 months after completing the CBIPE program. To improve the effectiveness of the writing, the authors could consider conducting a comparative study to provide a more in-depth analysis and evaluation of the CBIPE approach. This would help to establish a baseline for comparison and provide more robust evidence of the approach's effectiveness. Consider using alternative methods to evaluate collaborative skills, such as self-reported questionnaires or peer evaluations, to gain a deeper understanding of changes in collaborative skills over time. Additionally, conducting qualitative research may provide a deeper understanding of inter-professional teams rather than just generalizability. Another limitation of the study is that students' IPE competencies scale may be influenced by beliefs and attitudes factors, over which the authors had no control. Therefore, it is essential to investigate the ability to work as an active member of a multiprofessional team.

## Conclusions

This study highlights the positive impact of an educational intervention that aimed to teach and develop CBIPE courses for medical and health promotion students. Using a single-group, pre-posttest design, the study demonstrated that the intervention positively affected the improvement of IPE competency skills. However, managing scheduling, timetabling, and finding appropriate teaching resources for the increasing number of enrolled students can be challenging, and it requires coordinated efforts from the administration and faculty. It is also important to consider students' socio-demographic characteristics, cultural backgrounds, expectations, and attitudes toward their CBIPE experience and learning. These factors vary across countries and institutions and should be considered when interpreting the findings. Further analysis of clinical trials may help identify the CBIPE program's effect on the students' IPE competence.

## Data Availability

The data provided in this research can be obtained by contacting the corresponding author upon request. The data is not accessible to the public due to limitations related to privacy or ethical considerations.
